# Desalination of Municipal Wastewater Using Forward Osmosis

**DOI:** 10.3390/membranes11020119

**Published:** 2021-02-08

**Authors:** Elorm Obotey Ezugbe, Emmanuel Kweinor Tetteh, Sudesh Rathilal, Dennis Asante-Sackey, Gloria Amo-Duodu

**Affiliations:** Green Engineering and Sustainability Research Group, Department of Chemical Engineering, Faculty of Engineering and The Built Environment, Durban University of Technology, Durban 4001, South Africa; elormezugbe.ee6@gmail.com (E.O.E.); rathilals@dut.ac.za (S.R.); ingsackey@gmail.com (D.A.-S.); gamoduodu04@gmail.com (G.A.-D.)

**Keywords:** forward osmosis, membrane, municipal wastewater, salts, permeate

## Abstract

Membrane technology has gained much ground in water and wastewater treatment over the past couple of decades. This is timely, as the world explores smart, eco-friendly, and cheap water and wastewater treatment technologies in its quest to make potable water and sanitation commonplace in all parts of the world. Against this background, this study investigated forward osmosis (FO) in the removal of salts (chlorides, sulphates, and carbonates) and organics (chemical oxygen demand (COD), turbidity, total suspended solids (TSS), and color) from a synthetic municipal wastewater (MWW), mimicking secondary-treated industrial wastewater, at very low feed and draw solution flow rates (0.16 and 0.14 L/min respectively), using 70 g/L NaCl solution as the draw solution. The results obtained showed an average of 97.67% rejection of SO_4_^2−^ and CO_3_^2−^ while Cl^−^ was found to enrich the feed solution (FS). An average removal of 88.92% was achieved for the organics. A permeation flux of 5.06 L/m^2^.h was obtained. The kinetics of the ions transport was studied, and was found to fit the second-order kinetic model, with Pearson’s R-values of 0.998 and 0.974 for Cl^−^ and CO_3_^2−^ respectively. The study proves FO as a potential technology to desalinate saline MWW.

## 1. Introduction

Municipalities transport and treat large volumes of wastewater daily. It is reported that about 380 billion cubic meters of wastewater is produced annually worldwide [1]. In South Africa alone, approximately 7589 mega liters of wastewater is transported throughout the municipal sewers everyday [2]. These figures indicate the pressure placed on treatment facilities to ensure proper treatment and disposal of wastewater. Over the years, improvement in wastewater treatment in general became imperative because of environmental effects of wastewater discharge, the need for alternative source of water through wastewater treatment for reuse and the long-term effects of some specific constituents (like n-nitrosodimethylamine (NDMA), pesticides and phenolic compounds) of the wastewater causing neuroendocrine and mutagenic effects on aquatic life [3,4].

Characteristically, municipal wastewater (MWW) consists of a variety of contaminants. Because of its intrinsic nature of having different sources, municipal wastewater is highly complex and an efficient ready-to-go treatment method is still a challenge to arrive at. Specifically, inconsistent salt concentrations in MWW are reported from sources such as tanneries, textile industries, food processing, petroleum processing, and the chloroalkali chemical industries among other sources, which are destinations to at least 30 million tons of salt (NaCl) annually [5,6,7]. 

Many treatment methods have been utilized at different stages of MWW treatment for discharge and reuse. These include, but not limited to, conventional filtration processes, coagulation-flocculation, sedimentation, and biological treatment methods [8]. Advanced treatment methods include membrane processes and advanced oxidation processes. For the best part, most MWW treatment facilities employ sand and membrane filtration, chlorination and UV disinfection with the aim of removing turbidity, pathogens, and nutrients [3]. These processes, however, do not remove dissolved salts from the wastewater streams and these end up in the environment causing devastating effects on the aquatic system, agricultural lands, surface and ground water, and downstream water treatment facilities [9,10].

In a study conducted by Khong, et al. [11], it was observed that salinization of the Mekong River Delta due to different naturogenic and anthropic activities caused significant reduction in agricultural production and farm income in Vietnam leading to about 30% reduction in crop yield. In the same vein, exposure of agricultural lands to saline waters has been associated with long-term soil sodification, ground water salinization, and ion toxicity in plants, affecting the entire plant physiology and consequently leading to ill plant health [12,13]. Again, the ripple effects of salinization of ground water, due to a series of activities including inefficient wastewater treatment and discharge, on children were studied and found to have serious cognitive impairment consequences on them [14]. In the aquatic community, increasing salinity of fresh water bodies due to both natural and human activities has been identified as altering the growth and biochemical constituents of micro algae, amphibians, and other freshwater adapted species and this has dire consequences on the food chain within the aquatic ecosystem [15,16].

Desalination processes span from seawater and brackish water, to wastewater. The desalination process used is mainly dependent on the salinity of the feed being treated. In most cases, thermal desalination techniques like multistage flash (MSF) evaporation, and membrane processes like reverse osmosis (RO), nano filtration (NF) and electrodialysis are employed for reclamation of water through desalination [17]. These processes, however, are energy intensive, making reclaimed water expensive. 

In recent times, forward osmosis (FO), a potential energy saving desalination process, is being explored to optimize water recovery through simultaneous desalination and wastewater treatment. FO is an equilibrium-based, osmotically driven membrane separation process that has been used in several applications including concentration of specific feed streams, recovery of valuable nutrients, dilution of concentrated streams and desalination [18]. Table 1 shows some applications of FO.

Unlike the pressure-driven membrane processes, FO utilizes the difference in concentration of two solutions to cause the movement of water molecules from one point to the other. A draw solution (DS), which is the more concentrated solution, draws water from the feed solution (FS) across a semi-permeable membrane. Typical of the FO process, the DS soon becomes diluted and re-concentration of the DS is done to recycle the draw solutes as well as recover purified water [17,25,26]. The recovery and re-concentration process mainly depends on the type of DS used. This may include nanofiltration and/or reverse osmosis for salt-based draw solutes, thermal separation for gases and volatile compounds, or magnetic separation for magnetic nano-particles [27,28].

Aside from the advantages of the flexibility of operation, easy fouling reversal and energy utilization that FO brings, the process, including the reconstitution of DS, serves as a multi-barrier in rejecting salts and other contaminants [29,30]. The FO process also provides a viable option for seawater desalination when fertilizers such as NH_4_H_2_PO_4_, (NH_4_)_2_HPO_4_, Ca(NO_3_)_2_, and (NH_4_)_2_SO_4_ are used as draw solutions. When these DS (fertilizers) become diluted, they can be directly applied to crops without further treatment [31].

The main challenges with FO include the production of a suitable membrane for large-scale application of the process. A suitable FO membrane should have high permeability for water and low reverse solute flux (RSF). Also, it should be thin, mechanically strong, and able to resist internal concentration polarization (ICP). In this vein, much research has been conducted to improve FO membrane properties to enhance the process. FO membrane modifications have shown the potential of reducing ICP, fouling, and improving water flux [32,33]. Other forms of improvement of the FO process are in the membrane module development and draw solute improvement to reduce reverse solute flux. On the subject of FO, most studies have focused mainly on the concentration of FS with the aim of volume reduction for discharge or for resource recovery [34,35,36,37,38], at relatively high DS and FS flow rates (>1 L/min).

This study looked at the removal and feed concentration kinetics of chlorides, sulphates, and carbonates from municipal wastewater using 70 g/L NaCl as the draw solution at very low FS and DS flow rates (0.16 and 0.14 L/m, respectively). These salts are known to have negative effects on the environment when they get above the disposable limits. Again, their presence in reclaimed water causes scale and corrosion in pipes and water channels. In addition, chemical oxygen demand (COD), color, turbidity, and total suspended solids (TSS) were also monitored, as they form an integral part of MWW. The experiment holistically looked at the FO process from the determination of pure water flux to flux recovery through membrane cleaning. Each run was conducted in the continuous dilution mode for 6 h. To cater for repeatability, the experiment was conducted in triplicates. The concentrations of the targeted salts as well as conductivity of the system were monitored on an hourly basis. The permeate from this process can be recovered using reverse osmosis, which can also reconstitute the DS.

## 2. Materials and Methods

The set-up mainly consisted of two peristaltic pumps (Blue-White Industries, Huntington Beach, CA, USA) for circulation of FS and DS, a membrane test cell, flat sheet cellulose triacetate (CTA) membrane and two 5 L Duran bottles, used as feed and draw solution tanks.

### 2.1. CTA Membrane

CTA membrane with embedded support (Sterlitech, Kent, WA, USA), with properties given in Table 2, was used in this experiment. The membrane came as a square sheet of dimension 30.5 × 30.5 cm packed in 1% sodium metabisulfite water solution. Before use, the membrane was cut into the required dimension of 9 × 25 cm (effective membrane area of 0.0225 m^2^) and thoroughly rinsed with deionized (DI) water. It was then soaked in DI water overnight before use.

**Figure 1:**
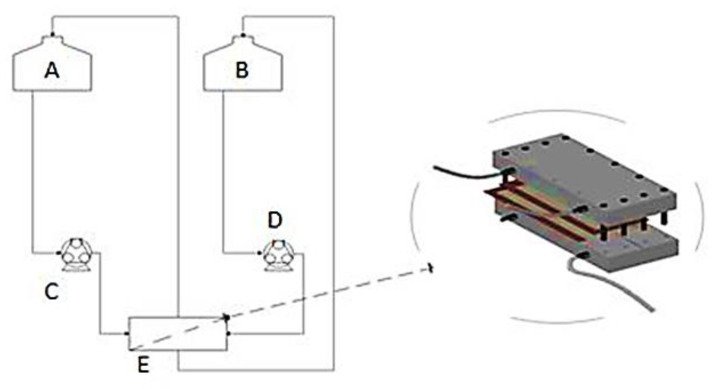
Process flow diagram (PFD) for FO process showing major equipment used: A = FS tank (5 L), B = DS tank (5 L), C = FS circulation pump, D = DS circulation pump, E = flat sheet membrane test cell.

The membrane test cell comprised of two PVC blocks of dimensions 35 cm × 15 cm × 6 cm, between which was sandwiched the CTA FO membrane and a plastic seal to avoid leakage. Rubber tubing was fitted onto nozzles that connected the test cell to the draw and feed solution tanks and the peristaltic pumps. Figure 1 shows the process flow diagram (PFD) of the setup. The feed and draw solutions were continuously stirred to enhance homogeneity, using two independent magnetic stirrers (Favorit, Selangor, Malaysia).

Feed for the experiment was simulated to mimic municipal wastewater having its main source from industry [39]. Table 3 shows the composition of the feed. All chemicals were of analytical grade and were homogeneously dissolved in 10 L deionized (DI) water (ELGA PURELAB Option-Q water deionizer, UK) at room temperature (20 ± 2 °C). The DS was prepared by dissolving 70 g NaCl (Sigma Aldrich, JHB, Malaysia), (osmotic pressure of 58.162 atm [31]) in 1 L DI water.

### 2.2. Process Description

The feed tank was filled with the feed solution (Table 4 shows the physicochemical parameters) to the 4.5 L mark while the draw solution tank was filled to the 1.5 L mark with the DS. The DS configuration adopted was the continuous dilution method, in which the draw solution was allowed to be diluted with water drawn from the feed for the entire duration of the experiment [19]. The membrane was oriented such that the active layer faced the feed solution. Counter current flow of DS and FS was used in this experiment [40,41]. FS and DS flow rates were maintained at 0.16 L/min (maximum flow rate of the pump) and 0.14 L/min (90% discharge rate of pump) respectively. The experiment took place at room temperature (20 ± 2 °C).

Effluent analysis was performed using Oakton EcoTestr™ pH1 Waterproof Pocket Tester for pH, HI98703-02, Turbidity meter (Hanna Instruments, Woonsocket, RI, USA) for turbidity and HI98130 pH&EC (Hanna Instruments, Woonsocket, RI, USA) for conductivity. COD, TSS, color, chlorides, carbonates, and sulfates were analyzed using DR 3900 Photometer (HACH, Loveland, CO, USA). All analyses were done in triplicates.

The set-up was left to run for 6 h after which component rejection, permeate flux, and reverse solute flux were determined according to the following formulas.

Since the permeate diluted the DS, the dilution factor (*D_f_*) was calculated as follows [42];

(1) $$\mathrm{Dilution}\;\mathrm{factor}\;(D_{f})=\frac{V_{f,DS}}{V_{p}}$$

where *V_f,DS_* is the final volume of the DS and *V_p_* is the volume of permeate.

(2) $$\mathrm{Component}\;\mathrm{Rejection}\;(\%)=\frac{C_{0}-D_{f}C_{f}}{C_{0}}\times 100$$

where *C*_0_ and *C_f_* are initial and final concentrations of the targeted component in the FS and DS, respectively, and *D_f_* is the dilution factor.

(3) $$\mathrm{Permeate}\;\mathrm{flux}\;(J)=\frac{\mathrm{Volume}\;\mathrm{of}\;\mathrm{permeate}\;(L)}{\mathrm{Effective}\;\mathrm{membrane}\;\mathrm{area}\left(m^{2}\right)\times \mathrm{time}\left(h\right)}$$

Volume of permeate was determined by taking the difference between the initial and final volumes of the draw solution.

(4) $$\mathrm{Reverse}\;\mathrm{solute}\;\mathrm{flux}\;(\mathrm{RSF})=\frac{C_{f}V_{f}-C_{0}V_{0}}{At}$$

where *C_f_* and *C*_0_ have their usual meanings and *V*_0_ and *V_f_* are the initial and final volumes of the FS respectively, *A* is the effective membrane area (m^2^), and *t* is the time (h).

### 2.3. Determination of Pure Water Flux

In order to assess flux decline and flux recoverability (after membrane cleaning), virgin membranes were subjected to an integrity test to determine the pure water flux. To this effect, three tests were conducted at the same conditions; 3 L of DI water was used as the feed solution while 1 L of 1 M NaCl solution was used as the draw solution [43]. The tests were conducted at room temperature (20 ± 2 °C). Each test lasted for 6 h.

### 2.4. Membrane Cleaning and Water Flux Recovery

The membrane was physically cleaned after each run. This was achieved by manually rubbing the membrane surface with fingers thoroughly under running water. After the second run, chemical cleaning was performed. First, DI waster was circulated at both FS and DS channels for 30 min. This was followed by 0.1% HCl solution circulated for 60 min. Further flushing was done using DI water to take away traces of the HCl solution.

Water flux recovery (WFR) was determined after the third (final) run according to Equation (5) [44];

WFR = (*J_c_*/*J*_0_) × 100 (5)

where, *J_c_* is the flux after membrane cleaning and *J*_0_ is the pure water flux.

## 3. Results and Discussion

### 3.1. Pure Water Flux

Table 5 presents the values obtained for the pure water flux (PWF). An average of 5.62 L/m^2^ h was achieved for all three runs. These values give an idea of the membrane’s performance in terms of permeation flux and serves as a benchmark to measure the efficiency of the membrane cleaning.

### 3.2. Permeation Flux

**Figure 2:**
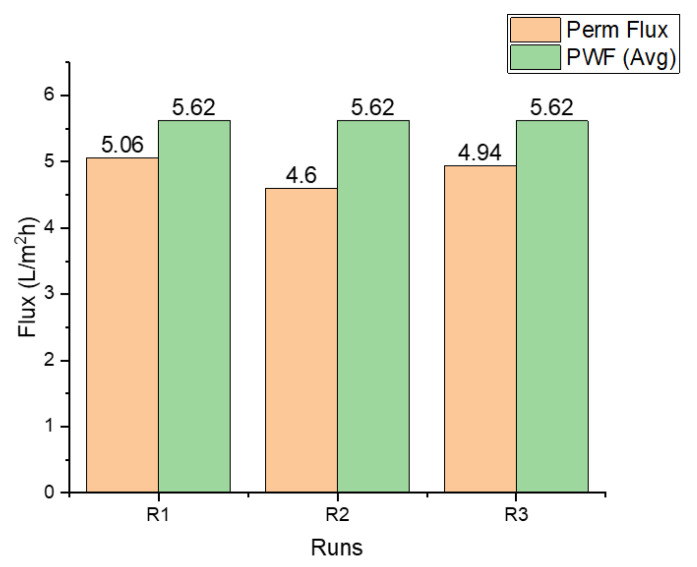
Permeate flux for each run (determined after 6 h), compared with the average pure water flux.

Water flux for the three runs is presented in Figure 2. Flux decline is apparent in the second and third runs most likely because of membrane fouling. A slight increase in flux in run 3 may be due to membrane cleaning performed after the second run. A combination of physical and chemical cleaning recovered 89% of flux. This was slightly above the performance of the physical cleaning alone, which was performed after run 1 with flux recovery of 82%.

Reverse salt flux (RSF) for the three runs is recorded in Table 6. Run 2 recorded the highest RSF, a significant increase from that of run 1. This could be due to accumulation of Cl^−^ ions within the membrane pores during the first run. This value decreased to 0.042 g/m^2^h after the third run because of the effectiveness of the chemical cleaning performed.

### 3.3. Rejection Efficiency of Membrane

**Figure 3:**
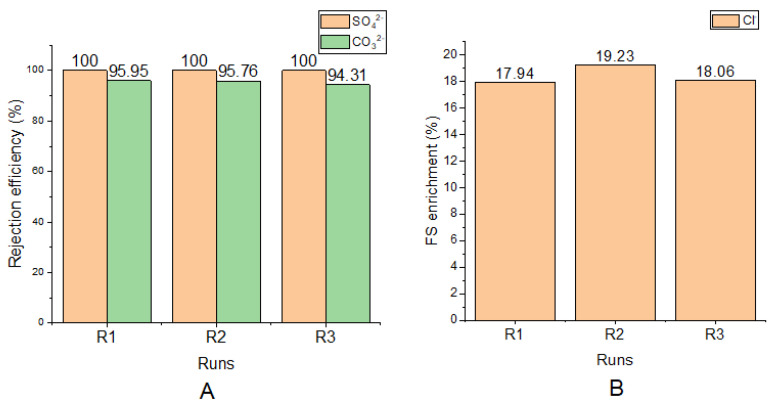
(**A**) SO_4_^2−^ and CO_3_^2−^ rejection efficiencies for each run; (**B**) Cl^−^ enrichment of FS (from DS) for each run.

The ion rejection efficiency of the FO membrane is shown in Figure 3. There was 100% rejection of SO_4_^2−^ in all runs, an average of 95.34 ± 0.74% rejection of CO_3_^2−^ in all runs, and an average of 18.41 ± 0.58% Cl^−^ enrichment of the FS in all runs.

The rejection efficiencies of the organic components (COD, TSS, color, and turbidity) are shown in Table 7 below.

The principal property of membranes used in separation applications is the ability to control the permeation of other species. For asymmetric FO membranes, this control is linked with the intrinsic membrane properties—water permeability (A), solute permeability (B), and the structural parameter of the support layer (S) [45]. In an experiment by Roest [46], which characterized the CTA membranes (the same used in this study), the A-value was found to be 1.4 LMH/bar, B-value was 0.74 µm/s, and S-value to be 1020 ± 60 µm.

With a solute permeation coefficient (B-value) of 0.74 µm/s, permeation of the solutes is highly limited. In addition to this, the mean pore size of the membrane, 0.74 nm [42] makes movement of solutes across the membrane extra restricted, especially for the divalent ions (SO_4_^2−^ and CO_3_^2−^) which have hydration radiuses larger than the mean pore size of the membrane. Another important factor known to play a role in rejection of solutes in membranes is the electrostatic interaction between the membrane and the solutes. At an approximate pH value of 4, the CTA membrane is isoelectric, above which the membrane becomes slightly negatively charged [42]. The working pH in this study was 6.7. This most likely may have triggered the negative nature of the membrane leading to the repulsion of the solutes. Combinations of these factors explain the rejection efficiencies listed in Table 7.

For all runs, Cl^−^, enriched the FS. This accounts for the negative values of 17.94, 19.23, and 18.06 that were recorded. These represent an increase in concentration of Cl^−^ in the FS. This movement of the Cl^−^ is due the chemical potential gradient between FS and DS, a phenomenon known as the reverse solute flux. When concentrations of solutes in the DS is higher than that of the FS, a backward movement of the solutes is triggered [47]. In this study, using 70 g/L NaCl as the draw solution makes available more Cl^−^ in the DS than in the FS. This was responsible for the backward movement. Again, the univalent nature of the Cl^−^ makes their penetration through the membrane easy.

### 3.4. Kinetic Studies of Cl^−^, SO_4_^2−^, and CO_3_^2−^ in FS

During FO, a lot of movement takes place in the system. The dynamics of the system depends on the process parameters like concentration, temperature, pH, and their changes with time. The kinetics of the targeted ions (Cl^−^, SO_4_^2−^, and CO_3_^2−^) in the FS was studied. This was achieved by the hourly monitoring of the concentration of these ions and the pH of the FS. The pH was found to be fairly constant, within the range 6.3–6.8, with an average of 6.62. The experiment was conducted at room temperature (20 ± 2 °C).

The change in concentration per hour was modelled according to second-order kinetics and was found to fit the model perfectly. The second order relates the concentration and time by Equation (6).

(6) $$\frac{1}{\left[C\right]}=kt+\frac{1}{\left[C_{0}\right]}$$

**Figure 4:**
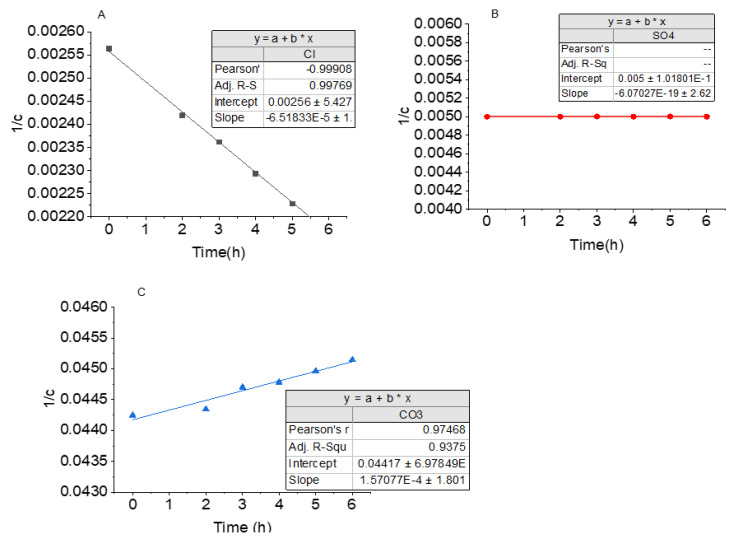
Second-order kinetic model for target ions, in the feed solution. (**A**) Negative slope due to steady increase in Cl^−^ concentration with time; (**B**) SO_4_^2−^ concentration remains the same in FS throughout the experiment; (**C**) CO_3_^2-^ concentration decreased with time, accounting for the positive slope.

The graphical representations, $\frac{1}{\left[C\right]}$ vs. *t*, of the kinetics is shown in Figure 4. In most cases, as the FO process proceeds, concentrated species in the FS is expected to reduce, as this depicts movement across the membrane.

The change in concentration of Cl^−^ with time is shown in Figure 4A. As time, *t*, increased, $\frac{1}{\left[C\right]}$ decreased almost linearly. Concentration of Cl^−^ was observed to be increasing in the FS as time elapsed. This is an indication of enrichment of Cl^−^ in the system and thus accounts for the negative rate constant (−6.518 × 10^−5^). The Pearson’s R-value of 0.998 was obtained, indicating the fitness of the model to the second-order kinetic model. With this, the concentration of the Cl^−^ in the feed can be predicted at a given time. Similar observations were made by other authors [37,48] in FO experiments using NaCl solution as the draw solution.

Change in concentration of CO_3_^2−^ over time is shown in Figure 4C. The positive rate constant (1.5707 × 10^−4^) depicts a reduction in concentration of CO_3_^2−^ with time. This reduction was, however, minimal, indicating a good rejection of the CO_3_^2−^ by the membrane. The Pearson’s R-value of 0.975, shows a good fitness to the second-order model, and can be used to predict CO_3_^2−^ concentration in the system at any given time. The trend of CO_3_^2−^ rejection/movement agrees with the rejection of divalent ions in the FO process observed by other authors [49,50].

Unlike the Cl^−^ and CO_3_^2−^, the graph of $\frac{1}{\left[C\right]}$ vs. *t*, for SO_4_^2−^ shows a straight line at a constant $\frac{1}{\left[C\right]}$ value of 0.005 (Figure 4B). This shows neither a reduction nor increase in the concentration of the SO_4_^2−^, which is an indication of a total rejection of SO_4_^2−^ by the membrane. SO_4_^2−^ and CO_3_^2−^ were similarly rejected in FO in previous studies [51].

A comparison of some previous studies of the application of FO in MWW desalination and treatment with the results of this study is shown in Table 8. In most cases, organic contaminant rejection was at least 70%, while salts rejection was above 90%, making this study consistent with results of previous studies [45,46,47].

## 4. Conclusions and Recommendations

In this study, simulated MWW, mimicking effluent from industrial processes was desalinated using forward osmosis. The study demonstrated that FO remains a promising desalination technique worth further exploration. This was shown in the efficiency of the removal of contaminants for, both salts and the organics from the MWW. Membrane properties, size exclusion, and electrostatic interaction played a vital role in the rejection of contaminants while osmotic pressure gradient drove the movement of water through the membrane. The kinetics study showed the behavior of the ions in solution as time proceeded. Knowledge of the kinetics coupled with the membrane parameters gives a clearer understanding of the rejection efficiency of the FO process for this study. The permeate from this process can be recovered using RO or NF. There is, however, need for further research in dealing with reverse solute flux which is a major challenge with the FO process.

## Figures and Tables

**Table 1:** Applications of forward osmosis in treatment of different wastewater streams. Adapted from [17].

Application	Draw Solute Used	Result	Reference
Raw municipal wastewater	NaCl, MgCl_2_	Up to 70% water recovery	[19]
Coke-oven wastewater	NaCl, MgSO_2_ and CaCl_2_∙H_2_O (0.4–2.5 M)	96–98% removal of cyanide, phenols and COD	[18,20]
Reduction in volume of gas field produced water	1 M NaCl	50% of volume reduced	[21]
Coal mine wastewater desalination	More saline mine waster	More than 80% of volume of mine water recovered	[22]
Sewage (primary effluent)	NaCl, MgCl_2_∙6H_2_O	Low water recovery due to internal concentration polarization and fouling	[23]
Domestic wastewater	NaCl (35 g/L)	Over 90% contaminant removal	[24]

**Table 2:** Properties of forward osmosis (FO) membrane used in this study.

Membrane Type	Flat Sheet with Embedded Support
Membrane material composition	Cellulose triacetate
Embedded support	Polymer mesh
Membrane thickness	0.09652 mm
Max Temperature	60 °C
Mean membrane pore size	0.307 ± 0.003 nm

**Table 3:** Composition of simulated municipal wastewater, spiked with Cl^−^, SO_4_^2−^, and CO_3_^2−^ salts [39].

Component	Amount (g)
Peptone	8
Glucose	5.5
NaHCO_3_	2.885
Urea	3.75
Meat Extract	12.5
K_2_HPO_4_	3.5
CuCl_2_·2H_2_O	0.5
CaCl_2_·2H_2_O	14.88
CaSO·2H_2_O	15.34

**Table 4:** Physicochemical characteristics of simulated municipal wastewater (MWW).

Parameter	Value
COD (mg/L)	3200
Turbidity (NTU)	70.5
Total suspended solids (TSS) (mg/L)	94
Colour (PtCo)	680
pH	6.7
Chloride (Cl^−^) (mg/L)	390
Carbonate (CO_3_^2−^) (mg/L)	22.5
Conductivity (mS/cm)	10.78
Sulphate (SO_4_^2−^) (mg/L)	200

**Table 5:** Pure water flux.

Run	Value (L/m^2^h)
1	5.65
2	5.45
3	5.75
Average	5.62 ± 0.123

**Table 6:** Reverse salt flux (of Cl^−^) for each run, determined after 6 h of experimental run.

Run	Value (g/m^2^h)
1	0.0057
2	0.361
3	0.042

**Table 7:** Removal efficiencies for the organic components of simulated MWW.

Parameter	Removal Efficiency (Avg) %
COD	60.10 ± 2.60
TSS	100.00
Color	97.22 ± 0.18
Turbidity	98.36 ± 0.11

**Table 8:** Comparative studies on FO desalination of MWW by other authors and this study.

Contaminants	DS Used	Contaminant Rejection, %	Ref
DOC *, nitrogen, and phosphorus	Synthetic seawater	94	[52]
SO_4_^2−^, PO_4_^3−^	NaCl, 35 g/L	88 and 95	[19]
COD	NaCl, 35 g/L	71.9	[53]
DOC	NaCl, 35 g/L	99	[54]
NH_3_, TKN, and orthophosphate	NaCl, 70 g/L	84.7, 85 and 99.6	[34]
Organics (COD, turbidity, TSS, color) and salts (SO_4_^2−^, CO_3_^2−^)	NaCl, 70 g/L	88.92 and 97.67	Current study

* DOC = Dissolved organic carbon.

## Data Availability

Not applicable.
